# Inactivating p53 is essential for nerve growth factor receptor to promote melanoma-initiating cell-stemmed tumorigenesis

**DOI:** 10.1038/s41419-020-02758-6

**Published:** 2020-07-20

**Authors:** Leiwei Jiang, Shibo Huang, Jieqiong Wang, Yiwei Zhang, Yuqing Xiong, Shelya X. Zeng, Hua Lu

**Affiliations:** 1 Department of Biochemistry and Molecular Biology, Tulane University School of Medicine, New Orleans, LA 70112 USA; 2 Tulane Cancer Center, Tulane University School of Medicine, New Orleans, LA 70112 USA; 3 Department of Dermatology, Guizhou Provincial People’s Hospital, Guiyang City, Guizhou Province China; 4 Clinical Pharmacology Institute, Nanchang University, Nanchang, Jiangxi 330006 China

**Keywords:** Melanoma, Tumour-suppressor proteins

## Abstract

Nerve growth factor receptor (NGFR, CD271, or p75NTR) is highly expressed in melanoma-initiating cells (MICs) and is critical for their proliferation and tumorigenesis, and yet the underlying mechanism(s) remain incompletely understood. We previously showed that NGFR inhibits p53 activity in a negative feedback manner in various cancer cells. Here we report that this feedback inhibition of p53 by NGFR plays an essential role in maintaining the sphere formation (stem-like phenotype) and proliferation of MICs, and in promoting MIC-derived melanoma growth in vivo. Knockdown of NGFR markedly reduced the size and number of spheroid formation of melanoma cells, which can be rescued by ectopically expressed NGFR. This reduction was also reversed by depleting p53. Consistently, knockdown of NGFR led to the suppression of MIC-derived xenograft tumor growth by inducing the p53 pathway. These results demonstrate that the NGFR-p53 feedback loop is essential for maintaining MIC stem-like phenotype and MIC-derived tumorigenesis, and further validates NGFR as a potential target for developing a molecule-based therapy against melanoma.

## Introduction

Melanoma is the most malignant and deadly cutaneous cancer in the world with more than 90,000 new cases per year in the USA recently^1^. It has been shown that melanoma-initiating cells (MICs) (also called melanoma stem cells (MSCs)^2,3^ play a pivotal role in metastasis and drug resistance of melanoma. A number of studies have linked the p53 pathway^4,5^ with these malignant phenotypes of melanoma. Although TP53 is the most frequently mutated gene in all types of human cancers^6–8^, its mutation is relatively rare in human melanomas^9–11^. This is partially due to the fact that MDM2 and MDMX (also called MDM4), two p53’s physiological feedback inhibitors, are highly expressed in melanomas. MDM2 and MDMX act as partners in a complex to bind to the N-terminal and C-terminal domains of p53, consequently inactivating the latter by mediating its ubiquitination and degradation, and inhibiting its transcriptional activity^11–13^. Remarkably, both of MDM2 and MDMX have been shown to be therapeutic target candidates for anti-melanoma therapies^11,13^. Our recent study revealed that this p53-MDM2 loop is regulated by a nerve growth factor receptor (NGFR and also called CD271 or p75NTR) in colon and lung cancer cells^14^. We showed that NGFR, in a negative feedback manner, suppresses p53 functions by directly inhibiting its transcriptional activity and assisting MDM2 in p53 degradation, consequently promoting the growth of human lung cancer cell-derived xenograft tumors^14^. Interestingly, NGFR has been shown to play a role in MIC renewal and proliferation^15,16^, as well as melanoma tumorigenesis and metastasis^15,17^.

NGFR is a 75 kDa single-transmembrane orphan receptor and is normally expressed in the central and peripheral nervous system^18^. For its physiological functions, it often partners with other receptors, such as TrkA, and is involved in a multitude of processes during neurogenesis, such as neural cell death, neuronal differentiation, neurite growth, and synaptic plasticity^18^. However, its level is also considerably high in several primary and metastatic human cancers^16,17,19^, including melanoma^16,17^. Earlier studies identified NGFR as a potential biomarker for MICs, as it was highly expressed in MICs and was important for MIC-derived tumor growth^15,20^. Later, NGFR was shown to be critical for melanoma metastasis^21,22^. Its high level was associated with melanoma progress in a clinical case study^23^, although another study suggested that its protein is unstable and thus might not be an ideal biomarker for human melanoma clinically^24^. Most recently, a clinical gene profiling study suggested that NGFR might play a divergent role in melanocyte and melanoma development through two different signaling pathways^17^. These studies highlight the importance of NGFR in MICs’ renewal, proliferation, and derived tumorigenesis. However, it remains largely elusive how NGFR executes its oncogenic role in melanoma development and metastasis. In others words, what is the biochemical and molecular mechanism(s) underlying the essential role of NGFR in MICs’ stem-like phenotype and corresponding tumor growth? Also specifically, is the ability of NGFR to inactivate p53 attributed to its role in promoting MICs’ spheroid formation in vitro and tumor growth in vivo?

In our attempt to address these tempting issues, we found out that NGFR can indeed promote MIC sphere growth and proliferation, as well as MIC-stemmed colony formation and tumor growth, by abating the p53 pathway. As detailed below, knockdown of NGFR reduced the number and size of MICs’ spheres and inhibited their proliferation and colony formation. These stem-like cancerous phenotypes were remarkably rescued by either overexpression of ectopic NGFR or depleting endogenous p53 via its short hairpin RNAs (shRNAs). Consistently, knockdown of NGFR led to the suppression of MIC-stemmed tumorigenesis in a xenograft tumor model via marked activation of p53 and its pathway. Hence, our results demonstrate the essential role of the NGFR-p53 feedback loop in maintaining MICs’ stem-like phenotypes and their ability to initiate melanoma growth in vivo. These results further validate NGFR as a possible drug target candidate for future development of anti-melanoma therapy.

## Results

### Isolation of NGFR highly expressed in melanoma sphere cells

**Fig. 1:**
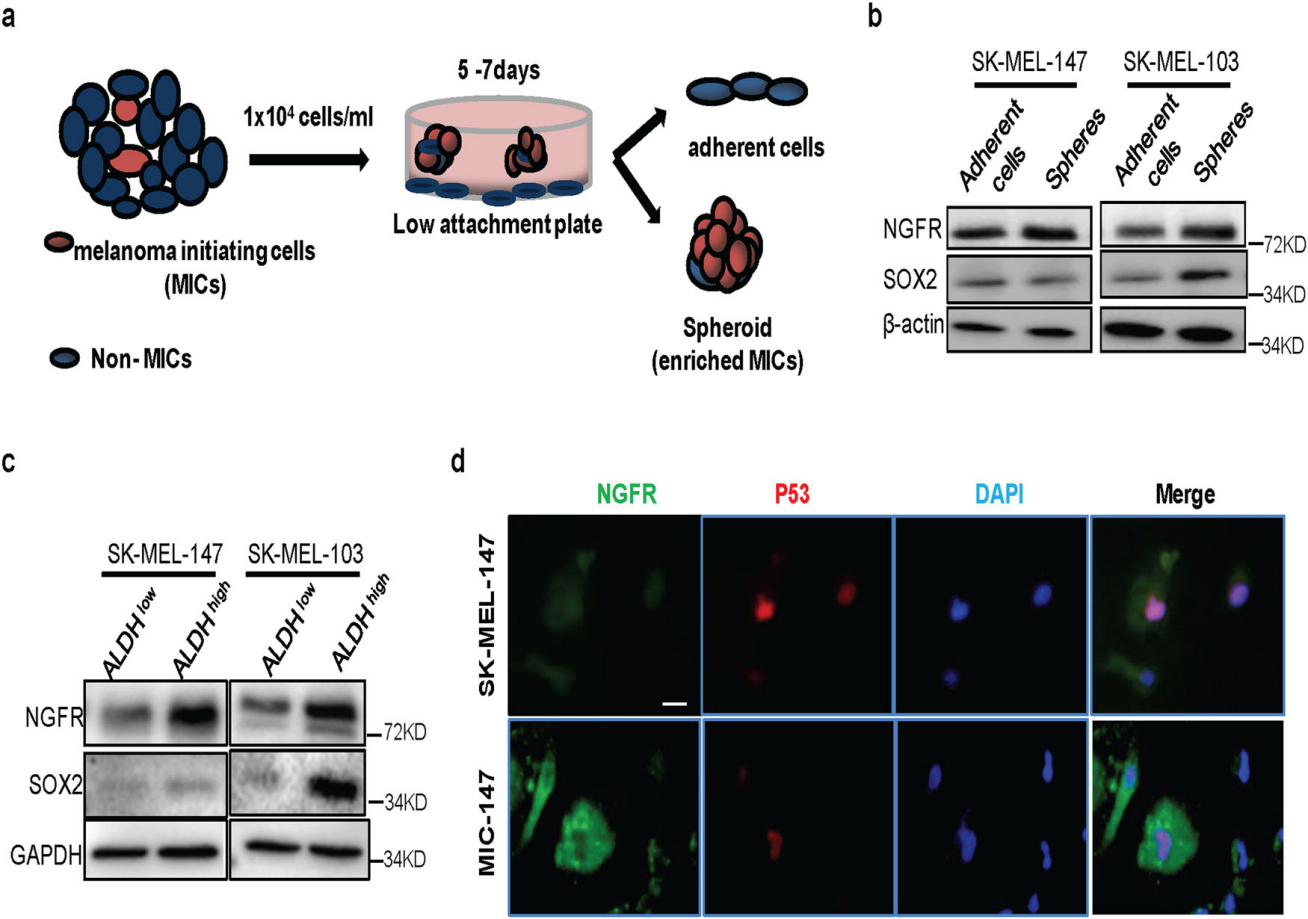
Establishment of MIC cells with high levels of NGFR and ALDH. **a** Schematic presentation of a non-adhesive culture system to enrich MICs from human melanoma cell lines. **b** Melanoma spheres express a higher level of NGFR proteins than do the adherent melanoma cells. Both of the adherent and sphere melanoma cells after selection were collected for WB analysis with indicated antibodies as described in the “Materials and Methods.” **c** FACS-sorted ALDH^high^ melanoma spheroids display stem cell-like features. ALDH^high^ and ALDH^low^ melanoma spheroids isolated from both SK-MEL-147 and SK-MEL-103 were collected for WB analysis with indicated antibodies. **d** NGFR is expressed much higher in MIC-147 cells than in SK-MEL-147 cells, compared with p53. Immunofluorescence (IF) staining analysis of SK-MEL-147 and MIC-147 cells was conducted with the anti-NGFR and anti-p53 antibody. IF images show cellular localization of NGFR (cytoplasm) and p53 (nuclei). Nuclei were counterstained with DAPI. Scale bar = 10 μm.

It has been shown that NGFR is highly expressed in MICs and is critical for their renewal and proliferation^15,16,20^. Our recent study revealed that by partnering with MDM2, NGFR can negate p53 activity in a negative feedback manner^14^. To determine whether this NGFR-p53 feedback regulation might play a role in MIC renewal and proliferation, we first isolated MIC cells from two human melanoma SK-MEL-147 and SK-MEL-103 cell lines, which harbor wild-type (wt) p53 by selecting aldehyde dehydrogenase (ALDH)-positive spheroid-enriched melanoma cells as described in the “Materials and Methods”^25,26^ (Fig. 1a). We then analyzed NGFR and SOX2 protein levels in both adherent and sphere melanoma cells by western blot (WB) analysis, as both of the proteins have been used as markers for MICs^15,27^. As shown in Fig. 1b, the NGFR protein level was markedly higher in sphere SK-MEL-147 and SK-MEL-103 cells than in adherent (non-sphere) SK-MEL-147 and SK-MEL-103 cells, although to a less degree in the latter cell line. However, the level of SOX2 was increased only in SK-MEL-103 cells but not in SK-MEL-147 cells (Fig. 1b, c). More markedly, the level of NGFR was even much higher in ALDH-high MIC cells than in ALDH-low MIC cells in both of the SK-MEL-147 and SK-MEL-103 cell lines after sorting ALDH-positive spheroid-enriched melanoma cells as described in the “Materials and Methods”^25,26^ (Fig. 1c), which we called MIC-147 and MIC-103 cells, respectively, in this manuscript. Again, SOX2 showed discrepancy in its protein level between MIC-147 and MIC-103 (Fig. 1c), suggesting that SOX2 might not be the downstream factor of NGFR in the MIC-147 cells. This NGFR result was further confirmed by immunofluorescence (IF) staining with the representative images for the SK-MEL-147 and MIC-147 cells (Fig. 1d), as NGFR signals were remarkably higher in MIC-147 than that in SK-MEL-147 cells. Interestingly, the intensity of p53 signals was inversely correlated with that of NGFR signals (Fig. 1d). This result is consistent with our previous results in other cancer cells^14^ and suggests that NGFR may regulate p53 level and activity in MIC cells as well, which will be tested below. These results demonstrate that we have isolated sphere-forming MIC cells from both of the melanoma cell lines, which contain high levels of ALDH and NGFR.

### NGFR is required for proliferation and clonogenicity of MICs

**Fig. 2:**
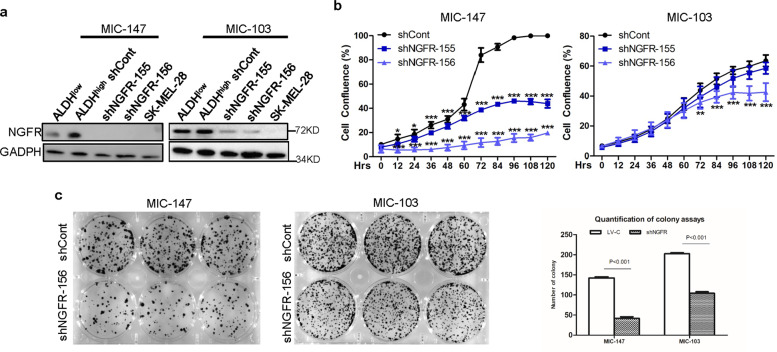
NGFR is required for proliferation and clonogenicity of melanoma-initiating cells. **a** shNGFRs effectively deplete endogenous NGFR in MICs isolated from both SK-MEL-147 and SK-MEL-103 cells as detected by WB analysis with indicated antibodies. **b** Knockdown of NGFR in two MIC clones reduces cell proliferation. MICs cells were transfected with shRNA targeting NGFR or control shRNA and then seeded in 96-well plates. Cell confluence (%) was calculated using Incucyte Zoom software based on phase-contrast images from 0 day to 5 days (data represent mean ± SD. *p < 0.05, **p < 0.01, ***p < 0.001 by one-way ANOVA). **c** NGFR knockdown inhibits clonogenicity of MICs. MIC-147 and MIC-103 cells isolated from SK-MEL-147 and SK-MEL-103 cells were transfected with shRNA targeting NGFR or control shRNA and seeded in six-well plates. Colonies were fixed by methanol and stained with crystal violet solution (left panel). Quantification of colonies is shown in the right panel (mean ± SEM, *n* = 3).

To test whether NGFR is essential for survival and proliferation of the MIC cells isolated above, we screened two sets of shRNAs specifically against NGFR using the lentivirus system and found that both of them can effectively reduce the protein level of endogenous NGFR in ALDH-high MIC-147 and MIC-103 cells with NGFR-deficient SK-MEL-28 cells as a control (Fig. 2a), compared with the control shRNA. Next, we determined whether NGFR knockdown might affect survival and proliferation of both of the MIC cells by conducting a set of cell growth assays as described in the “Materials and Methods.” As shown in Fig. 2b, shNGFR-156 more effectively suppressed survival of both MIC-147 and MIC-103 cells than did shNGFR-155, and also both of the shRNAs more effectively inhibited survival of MIC-147 than that of MIC-103 cells. Consistent with the result in Fig. 2b, knockdown of NGFR with the shNGFR-156 expression lentivirus significantly inhibited colony formation of both MIC-147 and MIC-103 cells (Fig. 2c, d). Taken together, these results indicate that NGFR is essential for proliferation, survival, and colony formation of MIC cells we isolated here, which is in agreement with previous studies^16,17^.

### Silencing of NGFR reduces melanoma stem-like sphere formation

**Fig. 3:**
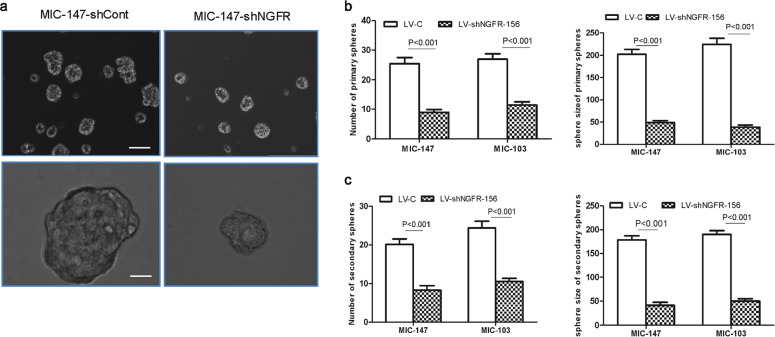
Silencing of NGFR reduces sphere self-renewal of melanoma-initiating cells. **a** Representative images as indicated show the reduction of the number and the size of MIC spheres after knockdown of NGFR. Scale bar = 150 μm. **b** The number (left panel) and the size (right panel) of primary MIC spheres decrease significantly after silencing of NGFR. **c** Reduction in the number (left panel) and the size (right panel) of secondary MIC spheres after knockdown of NGFR. The measurement of the number and size of secondary MIC spheres was conducted after transfection with LV-c, LV-shNGFR-156 in SK-MEL-147 and SK-MEL-103 cells. At least five independent fields of cells were counted and measured. The values represent the mean ± SEM of three independent assays.

Next, we determined whether NGFR is required for sphere formation of MIC cells. To do so, we used the shNGFR-156 lentivirus as described above (Fig. 2) to knock down endogenous NGFR in MIC-147 and MIC-103 cells, and conducted sphere formation analysis. Indeed, knockdown of NGFR significantly decreased the size and number of spheres of both MIC-147 and MIC-103 cells, compared with the control shRNA (Fig. 3b, c) with representative images of spheres for MIC-147 cells (Fig. 3a). This was true to both of primary (Fig. 3b) and secondary (Fig. 3c) sphere screenings. Also, we noticed that in our initial sphere screening with different melanoma cell lines, SK-MEL-28 cells that harbor mutant p53 (p53R145L mutant)^28^ and lack of NGFR (Fig. 2a) were unable to form spheres (data not shown), which were dramatically different from the NGFR-containing SK-MEL-147 or SK-MEL103 cells (Figs. 1–3). Together, these results demonstrate that NGFR is required for the stem-like sphere phenotype (self-renewal) of MICs.

### Knockdown of NGFR in MICs leads to activation of the p53 pathway

**Fig. 4:**
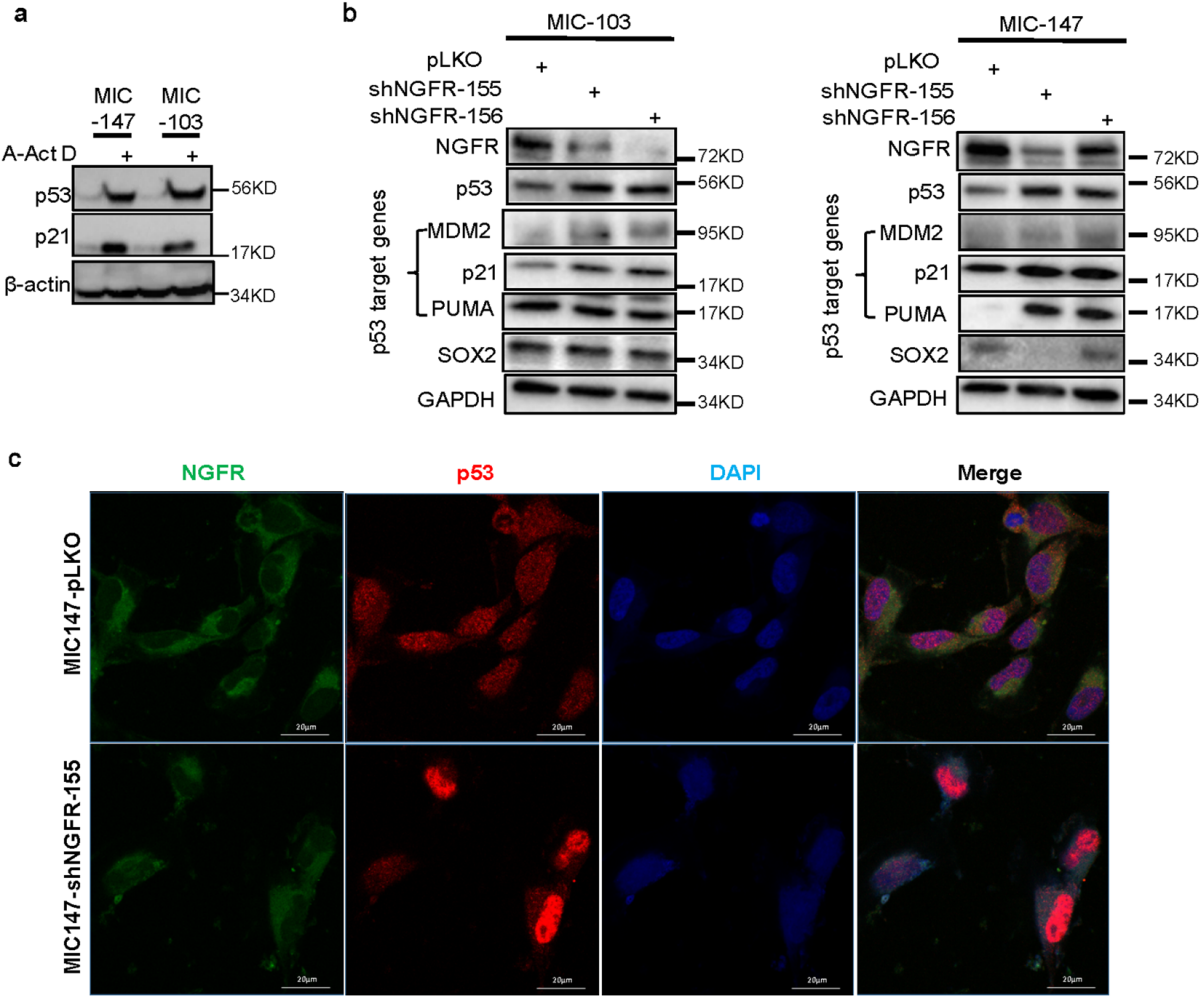
Knockdown of NGFR leads to p53 activation in melanoma-initiating cells. **a** Two MIC cell lines (MIC-147 and MIC-103) harbor wild-type p53. MIC-147 and MIC-103 cells after treatment with actinomycin D (Act D) were collected for WB analysis with antibodies as indicated. **b** NGFR knockdown induces p53 expression and activity. MIC-147 and MIC-103 cells were transfected with shRNA against NGFR or control shRNA followed by WB with antibodies as indicated. **c** NGFR knockdown induces p53 signals as detected by IF staining. MIC-147-shCont and MIC-147-shNGFR cells with spheroid phenotypes were collected for IF staining with antibodies and reagents as indicated. The images were captured under a confocal microscope, Scale bar = 20 μm.

We then tried to delineate the underlying mechanism(s) for how silencing NGFR leads to the dramatic reduction of sphere formation of MIC cells. NGFR has been shown to reduce wt p53 protein level by working with MDM2 and also to directly suppress p53’s transcriptional activity by binding to the central domain of p53^14^. First, we wanted to ensure that p53 is still in a wt form in MIC-147 and MIC-103 cells by examining its response to a DNA-damaging agent, as TP53 could be mutated during the selection of stem-like spheres from malignant melanoma cells that are inherited with incredible genomic instability. As shown in Fig. 4a, p53 and its target p21 were induced by actinomycin D in both of these MIC cells, indicating that p53 remains as wt in the sphere cells. Next, we determined if knockdown of NGFR might affect the levels of p53 and its downstream targets by using two different shRNAs against NGFR as described in Fig. 2 followed by WB analysis. Indeed, knockdown of NGFR by either of these shRNAs led to the elevation of p53 levels as well as the levels of its target gene-encoded proteins, such as MDM2, p21, and PUMA in both of MIC-147 and MIC-103 cells (Fig. 4b). This result was also confirmed by IF staining with anti-NGFR and anti-p53 antibodies, as the NGFR and p53 IF signals were inversely correlated with each other (Fig. 4c). These results demonstrate that depletion of endogenous NGFR results in p53 activation in MIC cells and suggest that activated p53 and its pathway might account for the drastic declining of the size and number of MICs’ spheres.

### Overexpression of NGFR rescues sphere and colony formation of NGFR-depleted MICs

**Fig. 5:**
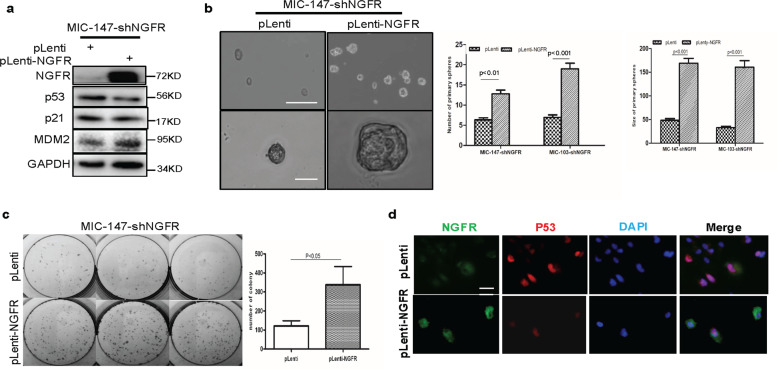
Overexpression of NGFR rescues spheroid formation and proliferation of NGFR-depleted melanoma-initiating cells. **a** Overexpression of NGFR in NGFR-depleted MIC cells leads to reduction of the p53 pathway. MIC-147-shCont and MIC-147-shNGFR cells were transfected with pLenti-NGFR, and 36 h after transfection, cells were collected for WB analysis with antibodies indicated. **b** Ectopic NGFR increases the size and number of spheres formed by scrambled shRNA-expressed and NGFR-depleted MIC cells. The same experiment as that shown in panel was conducted to analyze spheroid formation of the MIC cells. Representative images were taken with scale bar = 150 μm. The number (left panel) and the size (right panel) of primary MIC spheres for both MIC-147 and MIC-103 cell lines were quantified and presented in graphs after transfection of the MIC cells with pLenti or pLenti-NGFR. At least five independent fields of cells were counted and measured per condition, and in at least three independent experiments. This was true for the following experiments in Fig. 6 below. **c** Ectopic NGFR enhances clonogenicity of MICs. NGFR-depleted MIC-147 cells were transfected with pLenti or pLenti-NGFR and seeded in six-well plates. Colonies were fixed by methanol and stained with crystal violet solution (left panel). Quantification of colonies is shown in the right panel (mean ± SEM, *n* = 3). **d** IF analysis of the same cells as described in **c** was conducted with antibodies and reagents as indicated. Scale bar = 10 μm.

To ensure that impairment of the ability of MIC cells to form spheres and colony is due to the deficiency of NGFR control of p53, we tested whether overexpression of ectopic NGFR can rescue this impairment in the NGFR-depleted MIC cells. To do so, we introduced exogenous NGFR into NGFR-depleted MIC-147 or MIC-103 cells using the lentivirus expression system and carried out sphere formation, colony formation, and WB analyses. Ectopic NGFR caused the decrease of p53 and p21 levels, which were induced by knocking down endogenous NGFR in MIC-147 cells, as shown in the representative WB (Fig. 5a). Of note, MDM2 was slightly increased in the presence of ectopic NGFR, which might be due to the stabilization of MDM2 by NGFR via their direct binding. Correspondingly, ectopic NGFR also significantly increased the size and number of spheres of NGFR-depleted MIC-147 and MIC-103 cells (Fig. 5b). Furthermore, overexpression of ectopic NGFR drastically induced colony formation of these NGFR-deficient MIC cells as well (Fig. 5c). The reverse correlation of the exogenous NGFR level with the endogenous p53 level was evident in IF staining too. (Fig. 5d). These results demonstrate that loss of NGFR is the major cause for the impairment of stem-like sphere phenotype, colony formation, and survival of MIC cells after knocking down this protein. These results, consistent with that of Fig. 4, also suggest that p53 activation due to depletion of NGFR might be attributed to this impairment.

### Knockdown of p53 rescues sphere and colony formation of NGFR-depleted MICs

**Fig. 6:**
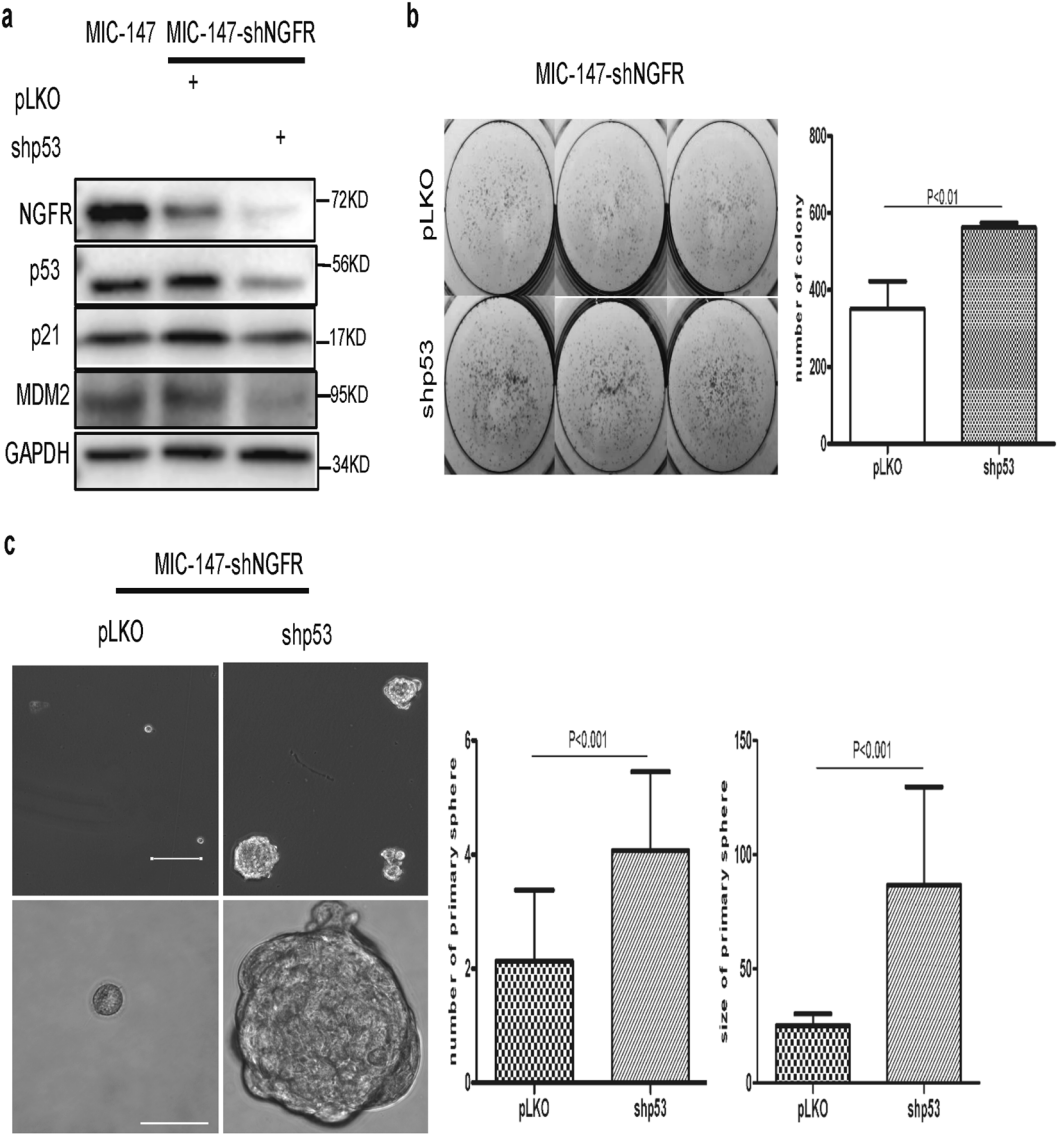
Knockdown of p53 rescues colony and spheroid formation of NGFR-depleted melanoma-initiating cells. **a** p53 knockdown reduces the protein levels of p53, p21, MDM2, and NGFR. NGFR-depleted MIC (MIC‐147‐shNGFR) cells were transfected with scramble and p53 shRNA independently. Cells were collected for WB analysis with indicated antibodies. **b** p53 knockdown enhances colonogenicity of NGFR-depleted MICs. MIC-147-shNGFR cells were transfected with pLKO or shp53 and seeded in six‐well plates. Colonies were fixed by methanol and stained with crystal violet solution (left panel). Quantification of colonies is shown in the right panel (mean ± SEM, *n* = 3). **c** p53 knockdown increases the size and number of NGFR-depleted MIC spheres. Representative images were taken after formation of spheres with the same cells as mentioned in **b**. Scale bar = 150 μm. The number (left panel) and the size (right panel) of primary MIC spheres are increased significantly after knockdown of p53. Count number and measurement of the size of the MIC cells were performed as described in Fig. 5b.

To determine whether activated p53 is indeed the prime molecular factor that causes the defects in sphere and colony formation of NGFR-depleted MIC cells, consequently suppressing the survival of these MIC cells, we also introduced shRNA specifically against p53 into these MIC cells and then performed the same set of experiments as described above. As expected^14^, knockdown of endogenous p53 not only reduced the levels of its targets p21 and MDM2, but further diminished the level of NGFR (Fig. 6a), which was previously identified as a transcriptional target of p53 as well^14^. Interestingly, the shRNA-mediated depletion of the p53 protein and its targets significantly enhanced colony (Fig. 6b) and sphere (Fig. 6c) formation of NGFR-silenced MIC-147 cells. Together with the results of Figs. 4 and 5, these results strongly validate that p53 activation as a result of NGFR knockdown largely account for the suppression of stem-like renewal (sphere phenotype), proliferation, colony formation, and survival of MIC cells. These results also strongly demonstrate that NGFR maintains the ability of MIC cells to sustain stemness-like phenotype (sphere formation) and proliferation by mainly inactivating p53 and its downstream pathway, and further indorse NGFR as a potential target for developing an anti-MIC agent useful for therapy against malignant and drug-resistant melanoma.

### Knockdown of NGFR abates MIC-stemmed tumorigenesis via p53 activation

**Fig. 7:**
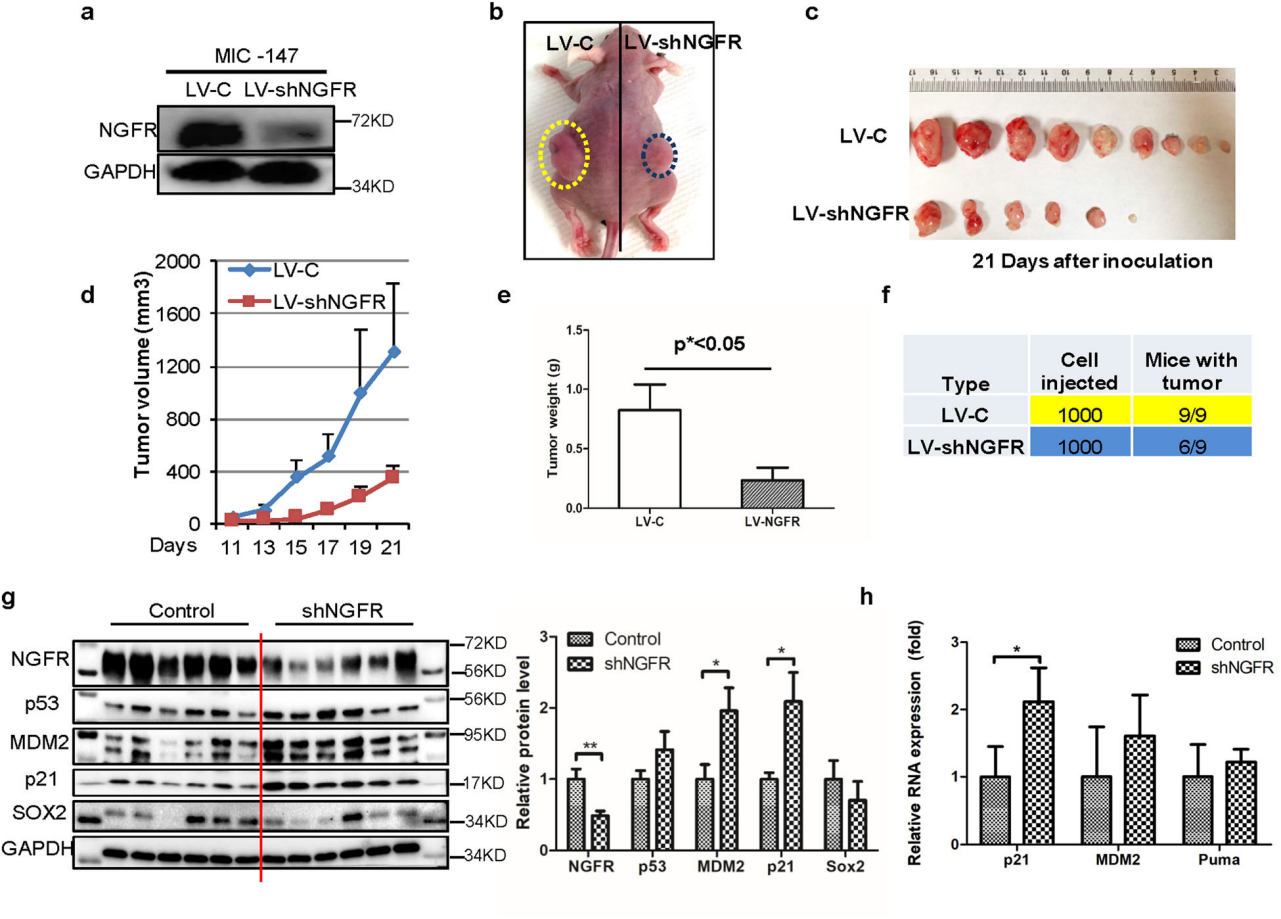
Knockdown of NGFR inhibits MIC-derived tumor growth in vivo. **a** Re-examination of NGFR levels in MIC cells used for xenograft tumors. WB analysis of NGFR levels was conducted with indicated antibodies in two MIC cell lines, MIC-147-control shRNA, and MIC-147-shNGFR, to confirm the knockdown efficiency of NGFR. **b** A representative image of subcutaneous xenografts in athymic nude mice 21 days after inoculation. **c** Xenograft tumor images showing that NGFR knockdown dramatically suppresses tumor growth in vivo after subcutaneous injection of 1000 MIC-147-LV-c or MIC-147-LV-shNGFR cells. **d** The tumor growth curve in volume. Tumor volumes were monitored during growth of MIC-derived xenograft tumors (mean ± SEM, *n* = 9). **e** A graph presenting the average tumor weight of each group as shown in **c** and **d**. **f** A table showing the tumor-initiation frequency in each group as shown in the above panels. **g** The p53 pathway is activated by NGFR knockdown in xenograft tumors. The protein levels of p53, p21, MDM2, and SOX2 were determined by WB with antibodies as indicated (mean ± SEM, *n* = 6). The quantification of each protein in both control siRNA and NGFR-depleted tumors is presented in a graph on the right. **h** The mRNA levels of p53 target genes in both control siRNA and NGFR-depleted xenograft tumors were detected by Q-PCR analysis. Data represent mean ± SEM. *p < 0.05, **p < 0.01 by two-tailed *t*-test.

Finally, we tested whether depleting NGFR might affect melanoma growth from MIC cells by activating p53. For this purpose, ~1000 NGFR-positive/ALDH-positive or NGFR-depleted/ALDH-positive sphere-forming MIC cells (Fig. 7a) as described above were subcutaneously injected into the flank of each nude mouse (Fig. 7b). Tumor growth was observed and measured every other day for 3 weeks as described in the “Materials and Methods,” and was then collected for further analyses. These MIC cells were highly tumor-prone, as within 3 days MIC-stemmed xenograft melanomas already started to grow, whereas the same number of non-MIC SK-MEL-147 cells took more than 2 weeks to begin to grow (Fig. S1b). Also, the average tumor weight of MIC tumors was 90% greater than that of non-MIC-tumors (Fig. S1a, c) by week 3. Interestingly, knockdown of NGFR significantly retarded the growth of MIC tumors and led to their drastic regression with the average tumor weight reduced by >70% compared with that of control MIC tumors in week 3 (Figs. 7c–e). More remarkably, three of nine NGFR-depleted MIC tumors did not grow at all (Fig. 7f). This regression of MIC tumors by depleting NGFR was well correlated with the activation of p53 and its pathway, as the protein and mRNA levels of p53 target genes, such as MDM2, p21, and PUMA, were induced along with the induced p53 level, and inversely proportional to the level of NGFR in these tumors (Fig. 7g, h). Although the protein level of SOX2, a marker of MSCs^27^, was not altered significantly, it showed the tendency of reduction in NGFR-depleted MIC tumors (Fig. 7g). In accordance with the results of Figs. 1–6, this result demonstrates that NGFR knockdown leads to the suppression of MIC-stemmed xenograft melanoma growth by activating the p53 pathway.

## Discussion

Although the biological role of NGFR in promoting MIC-stemmed melanoma development and metastasis has been established^17,21,22^ (regardless of the debate of whether it could serve as a useful marker for clinical melanoma diagnosis partially due to the instability of this protein^24^), the molecular mechanism(s) underlying this role remain largely obscure. In our attempt to address this question, our study as presented here revealed that primarily by inactivating p53, NGFR can maintain stemness-like spheroid phenotype of MICs and promote their proliferation and survival, as well as MIC-derived tumorigenesis (Figs. 1–7 and Supplementary Fig. S1). This conclusion is strongly supported by the following lines of evidence. First, in two of our established MIC cell lines, MIC-147 and MIC-103, knockdown of NGFR significantly reduced the size and quantity of stem-like spheres formed (Fig. 3), as well as their colony formation and cell survival (Fig. 2). Interestingly, this reduction was well correlated with the activation of p53 (Fig. 4), although without showing a clear change of SOX2 levels, a key factor in cancer stem cell renewal and proliferation, as well as a biomarker for MSCs^27,29^. Also, this reduction was completely rescued by overexpressing exogenous NGFR (Fig. 5), as well as by knocking down endogenous p53 in these NGFR-depleted MIC cells (Fig. 6). Finally, knockdown of NGFR significantly abrogated the growth of MIC-stemmed tumorigenesis by activating p53 (Fig. 7). Hence, our results strongly demonstrate that NGFR plays a crucial role in sustaining stem-like sphenoid formation, proliferation, survival, and tumorigenicity of MIC cells by negatively controlling p53 and its pathway. As the level of SOX2 was not elevated consistently between MIC-147 and MIC-103 cells, and when knocking down NGFR (Figs. 1 and 4), this suggests that SOX2 might not account for the proliferation and growth of MICs promoted by the activation of NGFR or NGFR inactivation of p53.

Our previous study showed that NGFR negates p53 functions through two mechanisms^14^. First, NGFR can bind to MDM2 and boost MDM2-mediated p53 ubiquitination and degradation. Also, NGFR can directly bind to the central domain of p53 and thus directly prevent its binding to its responsive DNA element at its target genes’ promoters, consequently inhibiting p53’s transcriptional activity^14^. These must also be the mechanisms underlying the inhibition of p53 by NGFR in MIC cells, because we observed the induction of p53 protein level (Figs. 4 and 7), but not its mRNA level (data not shown) by knocking down NGFR in MICs. In addition, we found that knocking down endogenous p53 in NGFR-depleted MIC cells further reduced the level of endogenous NGFR (Fig. 6), consistent with our previous result^14^. In line with this result, NGFR was hardly detectable in mutant p53-containing SK-MEL-28 cells (Fig. 2a; data not shown). Again, these results validate the NGFR gene as a p53 target gene^14^. In light of our previous study^14^ and the results as presented here (Figs. 1–7), we propose that NGFR negates p53 activity via a feedback manner in promoting MIC’s sphere formation and proliferation, as well as tumorigenesis. This negative inhibition of p53 by NGFR in MIC cells can also partially explain why aggressive and drug-resistant melanomas still sustain wt p53 in addition to high levels of MDM2 and MDMX.

Previous studies showed that targeting MDM2 or MDMX resulted in a therapeutic effect on killing melanoma cells and tumorigenicity^11,30^. MDMX often works together with MDM2 to enhance p53 degradation mediated by the latter, because MDMX, unlike MDM2, does not possess intrinsic E3 ubiquitin ligase activity^13,31^. As NGFR can partner with MDM2, but not MDMX, to inactivate p53 via ubiquitination-dependent proteolysis^14^, our findings as shown here also suggest that co-targeting MDMX or MDM2 and NGFR might serve as a better approach to more synergistically kill MIC cells. This combined strategy could be employed for the development of anti-MIC agents as a therapy against aggressive melanoma in the future. Thus, in conclusion, our studies as presented here not only elaborate the important role of the NGFR-p53 feedback loop in maintaining stem-like phenotype of MIC cells and boosting their proliferation, renewal, and survival, but also provide evidence for co-targeting NGFR and MDMX or MDM2 as a potential therapeutic strategy for highly aggressive and drug-resistant MIC-derived melanoma.

## Materials and Methods

### Cell lines and culture

The human melanoma cancer cell lines SK-Mel-103 and SK-Mel-147 were kindly provided by Dr. Shaomeng Wang at University of Michigan. SK-Mel-103 and SK-Mel-147 were cultured in Dulbecco’s modified Eagle’s medium (DMEM) supplemented with 10% fetal bovine serum (FBS) at 37 °C under a humidified 95 : 5 (%; *v*/*v*) mixture of air and CO_2_.

### Sphere formation culture system

SK-Mel-103 and SK-Mel-147 cells were independently cultured in DMEM supplemented with 5% FBS in ultra-low attachment plates (3261, Corning, USA). Cells were plated at a density of 1 × 10^4^ cells/ml and allowed to form spheroids for 5–7 days. Images were captured with digital inverted microscope (EVOS FL Cell Imaging System, Fisher Scientific).

### Aldefluor assay and fluorescence-activated cell sorting

The Aldefluor kit (Stem Cell Technologies, Vancouver, BC, Canada, http://www.stemcell.com) was used to profile and sort cells with high and low ALDH activity (ALDH^high^ and ALDH^low^), according to the manufacturer’s instructions. Melanoma spheres were collected and dissociated with trypsin to form a single cell suspension. The cell suspension was then treated with Aldefluor assay buffer containing ALDH protein substrate and incubated in a 37 °C water bath for 30 min. Sorting gates for fluorescence-activated cell sorting (FACS) were drawn relative to cell baseline fluorescence, which was determined by the additional treatment of ALDH-specific inhibitor diethylaminobenzaldehyde (DEAB) during the incubation. DEAB-treated samples were served as negative controls (ALDH-negative cells). ALDH^high^ and ALDH^low^ cells were sorted by a FACS flow cytometry (BD FACS Aria III) and analyzed in three independent experiments.

### Generation of stable MICs NGFR knockdown cell lines(MIC-147-shNGFR)

Lentiviral plasmids based on pLKO.1 system were packaged with the 2nd Generation Packaging System. Briefly, pLKO.1 vector containing scrambled or NGFR shRNAs, along with the packaging plasmids pMD2.G(VSVG) and pCMV-dR8.2, were transfected into HEK293T cells. The cells were maintained at 37 °C in a 5% CO_2_ humidified atmosphere for 72 h and the supernatant was collected to infect MIC-147, MIC-103 cells. The medium was changed by overnight infection. The infected cells were selected by 3 μg/ml puromycin for more than 7 days. The individual colonies from the transformation plate were analyzed.

### Generation of stably p53-knocked down MIC cells derived from NGFR-depleted MIC cell lines (MIC-147-shNGFR)

Lentiviral plasmids based on pLKO.1 system were packaged with the 2nd Generation Packaging System. Briefly, pLKO.1 vector containing scrambled or p53 shRNAs, along with the packaging plasmids pMD2.G(VSVG) and pCMV-dR8.2, were transfected into HEK293T cells. Cells were maintained at 37 °C in a 5% CO_2_ humidified atmosphere for 72 h and the supernatants were collected to infect MIC-147-shNGFR cells. Media were changed after overnight infection. Infected cells were selected with 3 μg/ml puromycin for more than 7 days. Individual colonies from the transformation plate were selected for further analyses.

### Generation of stably overexpressed NGFR MIC-147 cells derived from NGFR-depleted MIC cell lines (MIC-147-shNGFR)

Lentiviral plasmids based on pLenti6-V5 system were packaged with the 2nd Generation Packaging System. Briefly, pLenti6-V5 vector containing empty vector or NGFR, along with the packaging plasmids pMD2.G(VSVG) and pCMV-dR8.2, were transfected into HEK293T cells. The cells were maintained at 37 °C in a 5% CO_2_ humidified atmosphere for 72 h and supernatants were collected to infect MIC-147-shNGFR cells. The medium was changed after overnight culture. Infected cells were selected by 5 μg/ml blasticidin for more than 7 days. Individual colonies from the transformation plate were picked up for further analyses.

### Colony formation assay

Cells were trypsinized into single cell suspensions and seeded in six-well plates at a density of 1 × 10^3^ cells/ml. The medium was changed every 3 days until the colonies were visible. Colonies were fixed by methanol and stained with 0.5% crystal violet solution at room temperature for 30 min. After being destained with water, photographs were taken and clones were counted if they contained over 50 viable cells. ImageJ was used for quantification of the colonies.

### Cell proliferation assay

Cells were trypsinized into single cell suspensions and seeded in 96-well plates at a density of 2000 cells/well in Incucyte (IncuCyte® S3 Live-Cell Analysis System, Essen Biosciences). Images were obtained every 12 h using a ×4 objective for 5 days. Percentage of cell confluence was calculated using Incucyte Zoom software based on phase-contrast images.

### Self-renewal assay

The self-renewal assay was performed as previously described^32,33^, with some modifications. Briefly, MICs were stably infected with LV-c, LV-shNGFR156 to construct the stable NGFR knockdown cell lines and then stable NGFR knockdown cell lines were infected with overexpressed NGFR (LV-pLenti, LV-pLenti-NGFR) or knockdown p53(LV-pLKO, LV-pLKO-shp53). These stable cell lines were cultured in 60 mm low attachment plate at a density of 1 × 10^3^ cells/ml and let them to form primary melanoma spheres. After 1 or 2 weeks, the number and size of primary spheres were counted and measured. Then these primary melanoma spheres were dissociated and cultured using the same methods above. After another 1 or 2 weeks, secondary spheres were counted and measured. All the experiments were performed in triplicate and repeated at least three times.

### IF staining

Cells were fixed with 4% paraformaldehyde for 15 min at room temperature and permeabilized with 0.5% Triton solution for 15 min at room temperature. Cells were washed with phosphate-buffered saline, blocked with 8% bovine serum albumin (BSA), and incubated with primary antibodies (D4B3 for NGFR, 1:500 dilution; DO-1 for p53, 1:50 dilution) in 1% BSA in 4 °C overnight. Cells were then washed and incubated with the corresponding secondary antibodies and 4′,6-diamidino-2-phenylindole. Images were acquired using a fluorescence microscope (ZEISS, Axiovert 200 M) or a confocal microscope (ZEISS, AX10).

### Western blot analysis

WB analysis was carried out as previously described^14,34^. The following antibodies were used in these assays: NGFR (C-20, Santa Cruz Biotechnology and D4B3, Cell Signaling Technology, Danvers, MA, USA), SOX2 (D6D9, Cell signaling Technology, Danvers, MA, USA), p53 (DO-1, Santa Cruz Biotechnology), p21 (CP74, Neomarkers, Fremont, CA, USA), PUMA (H-136, Santa Cruz, Biotechnology), and MDM2 (SMP14, Santa Cruz Biotechnology, 2A9, 2A10, and 4B11).

### Reverse transcription and quantitative PCR analyses

Total RNA was extracted with TRIsure (Bioline, Luckenwalde, Germany) by following the manufacturer’s instructions. One microgram of total RNA was used as templates for reverse transcription using anchored Oligo (dT) 20 primer (Invitrogen) and M-MLV reverse transcriptase (Promega, Madison, WI, USA). Quantitative PCR (qPCR) was conducted using SYBR Green Mix according to the manufacturer’s protocol (BioRad, Hercules, CA, USA). The primers for human NGFR, p21, PUMA, and GAPDH were previously described^14^.

### Mouse xenograft experiments

Six-week-old athymic nude mice were purchased from Jackson Laboratories. Mice were subcutaneously inoculated with 1000 MIC-147 cells infected with lentivirus encoding control shRNA or NGFR shRNA in the left and right flanks, respectively. A total volume of 100 µL cells mixture with 1 : 1 growth factor-reduced Matrigel (BD Biosciences, San Jose, CA, USA) was injected each. Tumor growth was monitored every other day with electronic digital calipers (Thermo Scientific). Tumor volume was calculated with the formula: tumor volume (mm^3^) = length × width^2^ × 0.5. Mice were killed by euthanasia. Tumors tissues were excised, weighed, and snap-frozen in liquid nitrogen for WB and qPCR analysis.

### Statistical testing

All in vitro experiments were performed in biological triplicate and reproduced at least twice. The Student’s two-tailed *t*-test was used to determine mean difference among groups. *P* < 0.05 was considered statistically significant, asterisks represent significance in the following way: *p < 0.05; **p < 0.01. The term “n.s.” indicates that no significant difference was found. All the data are presented as mean ± SEM.

## Supplimentary information

Supplimentary information
Supplimentary Fig. 1
