# Supplementary material for: Inactivating p53 is essential for nerve growth factor receptor to promote melanoma-initiating cell-stemmed tumorigenesis
Source: Cell Death Dis. 2020 Jul 20;11(7):550. doi: 10.1038/s41419-020-02758-6 (PMC7371866; doi:10.1038/s41419-020-02758-6)
Supplement: Supplementary file 1 — Supplimentary information [file 41419_2020_2758_MOESM1_ESM.docx]

**Supplementary Information**

**Supplemental Figure Legends**

**Supplemental Figure 1. MIC-147 cells are much more cancer-prone than are their parental SK-MEL-147 cells.** (**a**) Comparison in size between MIC-tumors derived from MIC-147 cells and non-MIC-tumors derived from parental SK-MEL-17 cells. Tumors were isolated from xenograft melanomas at the time of sacrifice 18 days (MIC-147 cells) or 24 days (MEL-147 cells) post inoculation of 1000 MIC-147 cells or their parental SK-MEL-147 cells per mouse independently. (**b**) The growth curve of xenograft tumors in volume (n = eight mice per group). The error bars represent the SEM. Tumor initiated on day 18 in the SK-MEL-147 (M147) group. (**c**) The average weights of xenograft tumors at the endpoint in mice bearing either MIC-melanoma (MIC-147) or non-MIC-melanoma (MEL-147) tumors. Data represent mean ± SEM. ***p<0.001 by two tailed t-test.
