# Supplementary figures and images for: Inactivating p53 is essential for nerve growth factor receptor to promote melanoma-initiating cell-stemmed tumorigenesis
Source: Cell Death Dis. 2020 Jul 20;11(7):550. doi: 10.1038/s41419-020-02758-6 (PMC7371866; doi:10.1038/s41419-020-02758-6)

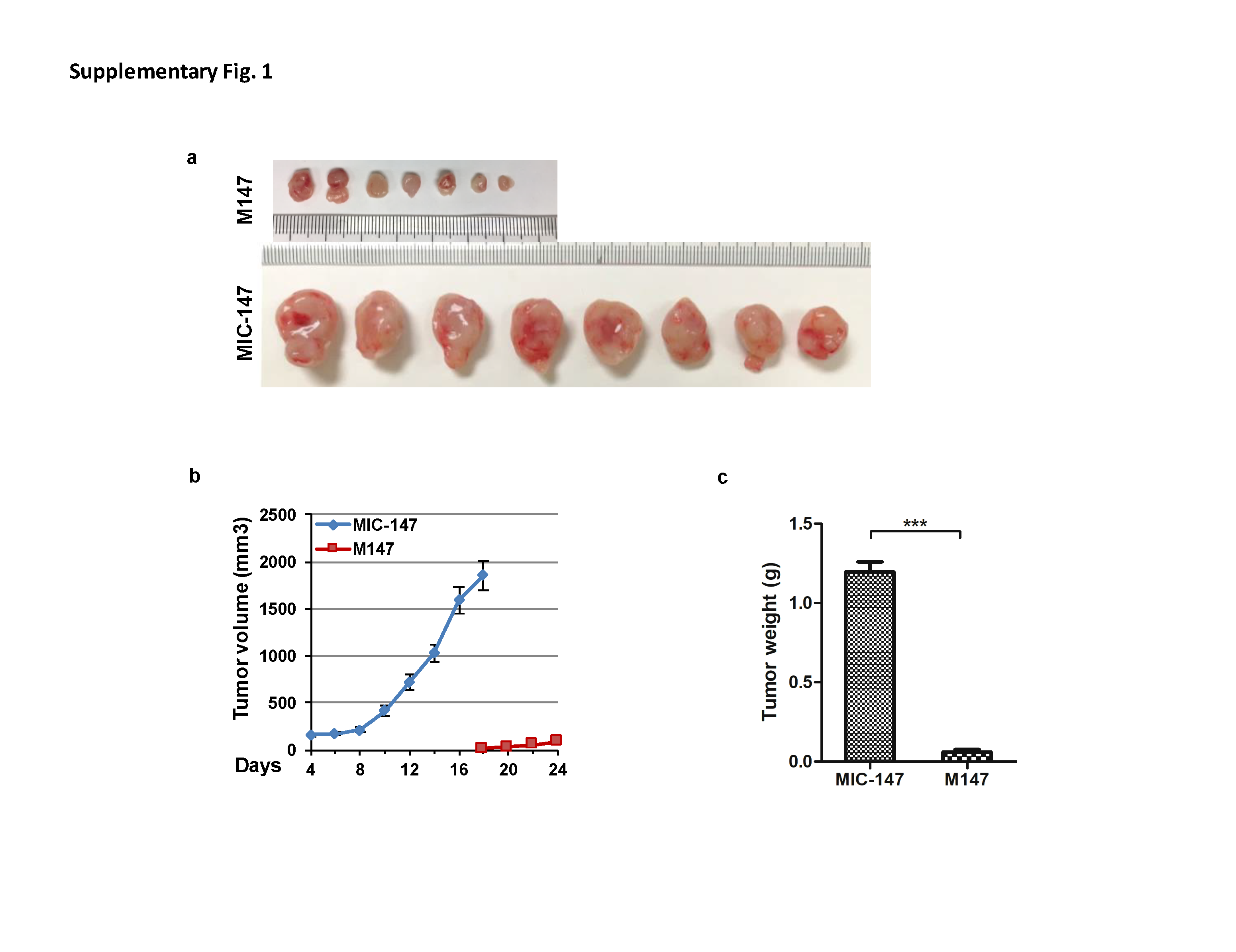

Supplement: Supplementary file 2 — Supplimentary Fig. 1 [file 41419_2020_2758_MOESM2_ESM.tif]
